# Historic data of the national electricity system transitions in Europe in 1990–2019 for retrospective evaluation of models

**DOI:** 10.1016/j.dib.2022.108459

**Published:** 2022-07-11

**Authors:** Marc Jaxa-Rozen, Xin Wen, Evelina Trutnevyte

**Affiliations:** Renewable Energy Systems, Institute for Environmental Sciences (ISE), Section of Earth and Environmental Sciences, University of Geneva, Switzerland

**Keywords:** Electricity system models, Energy scenarios, Model evaluation, Retrospective modeling, Ex-post, Hindcasting

## Abstract

This data package enables empirical analysis of national electricity system transitions and retrospective evaluation of electricity system models in 1990–2019 in 31 European countries, including the EU27, Switzerland, Iceland, Norway, and the United Kingdom. The data package covers two types of content. Firstly, we provide an annotated list of 528 original data sources and references relevant for retrospective electricity system modeling with emphasis on open-access sources. Secondly, we provide 1359 processed data files in a format that is suitable as input to electricity system models. Four types of data files are included for each country: (i) a country file documenting national electricity demand and economic data, (ii) technology files describing techno-economic data for each major generation technology in the country's electricity mix, (iii) resource files describing prices and CO_2_ emissions for each generation fuel or input resource, and (iv) load profiles describing 24 h national load curves for each available year. We provide these data files as comma-separated files to enable their wider reuse for retrospective evaluation of models as well as for empirical analyses of the European electricity system transitions.

## Specifications Table

**Table:** 

Subject	Energy (General)
Specific subject area	Empirical research on national electricity system transitions and retrospective evaluation of electricity system modeling in European countries
Type of data	Tables (annotated reference list and processed data files)
How the data were acquired	Literature review
Data format	Excel .xlsx file (annotated reference list) Comma-separated values (CSV) files (processed data files)
Description of data collection	The original data sources were acquired using a literature review with the emphasis on open-access sources, and these sources were then synthesized and harmonized to be usable in electricity system models as input parameters. All original data sources are referenced in the "Reference" column of the final processed data files. These sources are summarized in Table 2 of the article and detailed in an annotated reference list included as an Excel file in the data repository. Processing and harmonization steps are described in the main text of the article. Electricity demand load values for 1995–2005 were primarily digitized from reports in paper or in PDF, using optical character recognition, then manually verified for consistency.
Data source location	The processed data files cover 31 European countries: the EU27, Switzerland, Iceland, Norway, and the United Kingdom. The reference list includes a limited number of sources for other countries not in the main sample (Bosnia and Herzegovina, Serbia, Russia).
Data accessibility	Repository name: Zenodo Data identification number: DOI: 10.5281/zenodo.6338417 Direct URL to data: https://zenodo.org/record/6338417

## Value of the Data

• This data package synthesizes 528 original data sources for retrospective modeling or empirical analysis of the national electricity system transitions in 31 European countries in 1990–2019.
• The data package includes digitized historical electricity demand statistics that were previously unavailable in a machine-readable format.
• This data package enables transparent and replicable research on historical electricity system transitions and retrospective evaluation of models.

## 1 Data Description

**Fig. 1:**
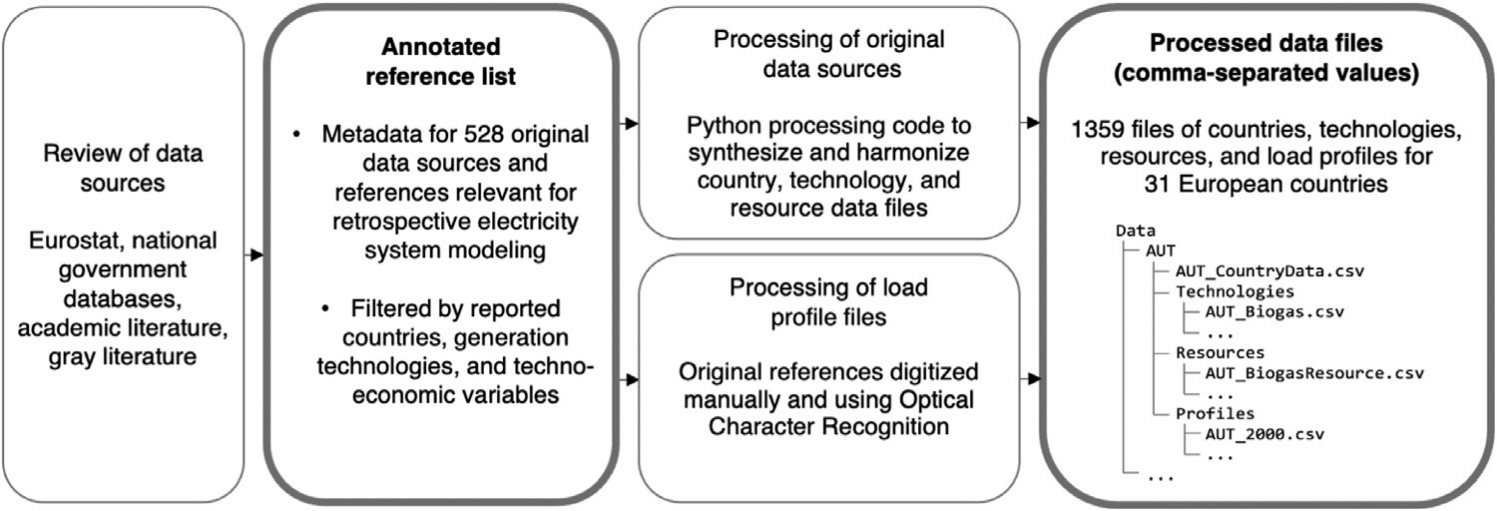
Data collection and processing steps. Steps outlined in bold denote files included in this data package.

Fig. 1 summarizes the collection and processing of the data package reported in this article. The data package covers two types of content: an annotated list of 528 original data sources and references, and a set of 1359 processed data files in a format that is suitable as input to electricity system models.

### 1.1 Annotated reference list

The annotated reference list provides metadata for the original data sources and other references relevant for the empirical analysis and retrospective modeling of national electricity system transitions in 31 European countries in 1990–2019. Metadata are comprised of bibliographic data, the country or countries described in the reference, the generation technologies and techno-economic variables described, and a brief description of the content (Table 1). The data can be filtered by countries, generation technologies, and techno-economic variables using MS Excel's filter search functionality, using unique values shown in Table 1.

**Table 1:** Countries, technologies, and techno-economic variables included in the annotated reference list.

Countries (ISO 3166-1 codes) and country groupings	AUT, BEL, BIH, BGR, CHE, CYP, CZE, DEU, DNK, ESP, EST, FIN, FRA, GBR, GRC, HUN, HRV, IRL, ISL, ITA, LTU, LUX, LVA, MLT, NLD, NOR, POL, PRT, ROU, RUS, SRB, SVK, SVN, SWE, EU, OECD, various, generic

Generation technologies	Coal, lignite, shale, peat, CCGT, OCGT, natural gas (general), oil, nuclear, CHP, biomass, biogas, landfill gas, waste incineration, offshore wind, onshore wind, PV, CSP, small hydropower, run-of-river hydropower, hydropower dam, pumped hydropower storage, hydropower (general), geothermal, import, various

Techno-economic variables	Installed capacity, annual generation, investment cost, decommissioning cost, levelized cost, fuel cost, fuel CO_2_ emissions, fixed O&M, variable O&M, levelized O&M, heat price, power price, load factor, thermal efficiency, heat-to-power ratio, own use, ramp rate, lifetime, build rate, peak load, base load, exchange rate, country GDP PPP per capita, country GDP deflator

*Note:* CCGT: combined cycle gas turbine; OCGT: open cycle gas turbine; CHP: combined heat and power; PV: photovoltaics; CSP: concentrated solar power; O&M: operation and maintenance; GDP: gross domestic product; PPP: purchasing power parity.

### 1.2 Processed Data Files

Processed data files include data files on countries, technologies, resources, and load profiles for each of the 31 countries. The files are formatted for use with the D-EXPANSE electricity sector modeling framework [1], but they are provided in a human-readable and machine-readable format to enable their wider reuse. The country, technology, and resource files were processed and harmonized using Python processing code and they use a "long" structure, reporting all variables as time series for 1990–2019. Table 2 summarizes the contents of the files and the main data sources used for each variable; specific references and any additional notes are listed in the "Reference" and "Note" columns of the processed data files. Where applicable, processing and harmonization steps for each variable are described in *Experimental design, materials and methods*. Values that were obtained from commercial sources and cannot be redistributed are replaced with blank values in the data files and can be found in the original sources. Alternative open-access data sources are suggested in the ``Note'' column when available. Load profile files were digitized and compiled separately and use a "wide" structure with separate files for each available year, containing columns of hourly load values and rows of days.

**Table 2:** Summary of data files, variables, and main data sources.

Type of data file	Variable name in data file	Variable description	Main data source
Country	ElSupplied_annual_central	Annual supplied electricity	Eurostat datasets [2] nrg_ind_peh (Gross and net production of electricity), nrg_bal_c (Complete energy balances)
	PeakDem_fromzero_central	Annual peak load	IEA Electricity Information [3]; UCTE Statistical Yearbook [4] (1995-2005); ENTSO-E Power Statistics [5] (2006-2014); Open Power System Data [6] (2015-2019)
	BaseDem_central	Annual base load	UCTE Statistical Yearbook [4] (1995-2005); ENTSO-E Power Statistics [5] (2006-2014); Open Power System Data [6] (2015-2019)
	Capacity_margin	Capacity margin	Own assumption for modeling
	Actual_import	Annual electricity imports	Eurostat dataset [2] nrg_bal_c
	Actual_export	Annual electricity exports	Eurostat dataset [2] nrg_bal_c
	Distribution_losses	Annual electricity distribution and transmission losses	Eurostat dataset [2] nrg_bal_c
	Export_profit	Export electricity price	Eurostat datasets [2] DS-018995 (EU trade since 1988 by SITC), nrg_bal_c, nrg_pc_205_h (Electricity prices for industrial consumers - bi-annual data, 1990-2006), nrg_pc_205 (Electricity prices for non-household consumers - bi-annual data, 2007-2019)
	Heat_revenue	CHP heat credit	Energiforsk [7]; OECD/Nuclear Energy Agency [8]
	Elasticity_factor	Price elasticity of electricity demand	Labandeira et al. [9]
	Discount_rate_uniform	Discount rate	Own assumption for modeling
	GDP_per_capita	GDP per capita (PPP)	World Bank dataset [10] NY.GDP.PCAP.PP.KD (GDP per capita, PPP)
	Population	Population	Eurostat dataset [2] tps00001 (Population, national level)

Technology	Resource	Resource file used by the technology	Own assumption for modeling
	Actual_generation	Annual generation	Eurostat datasets [2] nrg_ind_pehnf (Gross production of electricity and derived heat from non-combustible fuels), nrg_bal_c
	Actual_capacity	Generation capacity	Eurostat datasets [2] nrg_inf_epc (Electricity production capacities by main fuel groups); nrg_inf_epcrw (Electricity production capacities for renewables and wastes); Global Energy Monitor [11,12]; Eurelectric [13]
	Actual_new_capacity	New generation capacity	Computed from generation capacity time series
	Actual_retired_capacity	Retired generation capacity	Computed from generation capacity time series
	Initial_retired_capacity	Retired capacity existing in 1990	Computed from generation capacity time series
	Fuel_efficiency	Fuel efficiency of generation	Eurostat dataset [2] nrg_bal_c
	Own_use	Own use	Eurostat dataset [2] nrg_ind_peh
	Heat_to_electricity	Heat-to-power ratio of generation	JRC-IDEES database [14]; Eurostat dataset [2] nrg_bal_c
	Potential_installed	Technical potential of installed capacity	Tröndle et al. [15]
	Potential_annual	Technical potential of generation	Tröndle et al. [15]; Hoes et al. [16]; Stocks et al. [17]; JRC-ENSPRESO database [18]
	LF_max	Maximum annual load factor	Renewables.ninja [19,20]; ENTSO-E Pan-European Climatic Database [21]
	LF_min	Minimum annual load factor	ENTSO-E Pan-European Climatic Database [21]
	Ramp_rate	Ramp rate	Own assumption for modeling
	Peak_contr	Contribution of installed capacity to the peak load equation	Own assumption for modeling
	Inv	Investment cost	OECD/Nuclear Energy Agency [8]; IRENA [22]; EC-ASSET database
	Fixed_OM_annual	Fixed operation and maintenance cost	OECD/Nuclear Energy Agency [8]; EC-ASSET database [23]
	Variable_OM	Variable operation and maintenance cost	OECD/Nuclear Energy Agency [8]; EC-ASSET database [23]
	Learning_rate	Technology learning rate	Rubin et al. [24]
	Buildrates	Maximum annual addition of generation capacity	Own assumption for modeling
	Lifetime	Lifetime of generation capacity	Own assumption for modeling

Resource	Fuel_cost_fuel	Price of generation fuel or resource	IEA Energy Prices and Taxes [25]; JRC-ENSPRESO database; Eurostat datasets [2] DS-018995, nrg_bal_c, nrg_pc_205_h, nrg_pc_205
	CO2_generation	CO_2_ emissions of generation fuel or resource (per unit of fuel or resource)	IPCC [26]; Eurostat datasets [2] nrg_bal_c and nrg_ti_eh; European Environment Agency [27]

Load profile	N/A	Hourly load	UCTE Statistical Yearbooks [4] (2000-2005); ENTSO-E Power Statistics [5] (2006-2015); Open Power System Data [6] (2015-2019).

## 2 Experimental Design, Materials and Methods

The following sections summarize key references and assumptions used for processing the four types of data files from Table 2. The "Reference" column of the CSV data files lists specific references for each variable, and the "Note" column details additional processing and assumptions where applicable. We here provide a general reference to the Eurostat data portal which can be used to access specific referenced datasets with the Data Browser tool.

### 2.1 Country Data File

*Annual supplied electricity*: Annual supplied electricity is taken as the sum of domestic net generation (Eurostat nrg_ind_peh dataset [2], balance NEP) and net imports (Eurostat nrg_bal_c dataset, balance IMP).

*Annual peak load*: Peak load data is primarily taken from UCTE Statistical Yearbooks [4] (1995–2005 period), ENTSO-E Power Statistics [5] (2006–2014 period), and Open Power System Data [6] (2015–2019 period). Peak load data for 1990–1994 is taken from IEA Electricity Information [3] when available. Values for missing years are estimated using the average annual system's load factor in the next five-year period for which data is available.

*Annual base load*: Base load data is primarily taken from UCTE Statistical Yearbooks [4] (1995–2005 period), ENTSO-E Power Statistics [5] (2006–2014 period), and Open Power System Data [6] (2015–2019 period). Base load for missing years is estimated using the average fraction of base load relative to peak load in the next five-year period for which data is available.

*Capacity margin*: Modeling assumption.

*Annual electricity imports*: Eurostat nrg_bal_c dataset (balance IMP).

*Annual electricity exports*: Eurostat nrg_bal_c dataset (balance EXP).

*Annual electricity distribution and transmission losses*: Eurostat nrg_bal_c dataset (balance DL).

*Export electricity price:* Annual average export electricity prices are estimated by dividing value of exported electricity (Eurostat dataset DS-018995, SITC code 35) by the volume of exports (Eurostat dataset nrg_bal_c, balance EXP). Missing years are estimated using the average industrial electricity price in neighboring countries from Eurostat datasets nrg_pc_205_h and nrg_pc_205 (before 2007: price for demand band Ie, first semester; 2007 and later: band Id, first semester).

*CHP heat credit:* National heat price time series from Energiforsk [7] are indexed to 2015 OECD/NEA estimate for the credited value of CHP heat in OECD countries [8] (45 USD/MWh), assuming this estimate is representative of Germany. Time series for missing countries are estimated using the average Energiforsk value across all countries. Missing years are estimated by linearly interpolating available data points, then by back-filling and forward-filling remaining missing years.

*Price elasticity of electricity demand*: Assumed constant, based on Labandeira et al. [9]

*Discount rate*: Modeling assumption.

*GDP per capita*: World Bank World Development Indicators [1], dataset NY.GDP.PCAP.PP.KD (GDP per capita, PPP).

*Population*: Eurostat dataset tps00001 (Population, national-level).

### 2.2 Technology Data Files

*Annual generation*: Values are directly taken from Eurostat datasets nrg_bal_c for combustible fuels, and nrg_ind_pehnf for non-combustible fuels (balance GEP in both datasets). Combustible fuels are aggregated using the Standard International Energy Product Classification (SIEC) codes listed in the "Note" column of the CSV files.

*Generation capacity*: For coal, lignite, and natural gas generation, Global Energy Monitor plant-level datasets [11,12] are converted to time series of installed generation capacities using plant construction and retirement dates, assuming that plants with missing construction years were operational in 1990. Nuclear capacity is directly taken from Eurostat nrg_inf_epc dataset. Renewable generation capacities are directly taken from Eurostat nrg_inf_epcrw dataset. In case of inconsistency between installed capacity and annual generation (e.g. zero reported capacity with a certain amount of annual generation), capacity is adjusted as specified in the "Note" column because annual electricity generation data is assumed to be the most reliable.

*New generation capacity*: Computed from annual changes in generation capacity.

*Retired generation capacity*: Computed from annual changes in generation capacity.

*Retired generation capacity existing in 1990*: Computed from annual changes in generation capacity and the initial capacity assumed for 1990.

*Fuel efficiency of generation*: Fuel efficiency of thermal generation is taken as the gross electricity production from each fuel (Eurostat nrg_bal_c, balance GEP) divided by the total input of fuel for electricity and heat generation, excluding heat-only plants (Eurostat nrg_bal_c, balances TI_EHG_MAPE_E, TI_EHG_MAPCHP_E, TI_EHG_APE_E, TI_EHG_APCHP_E). To remove possible outliers, values are bounded to a range of 10–60%.

*Own use*: The fraction of own electricity use for each technology is estimated using time series for net and gross generation (Eurostat nrg_ind_peh, balances GEP and NEP).

*Heat-to-power ratio of generation*: Heat production for sale to third parties is estimated using total heat production from CHP plants (Eurostat nrg_bal_c, balances GHP_MAPCHP and GHP_APCHP), multiplied by the fractional share of small and extra-small CHP capacity in total CHP capacity in the JRC-IDEES database [14] for each generation technology. The result is divided by annual generation to obtain the heat-to-power ratio. To remove possible outliers, the heat-to-power ratio is adjusted to yield a maximum combined plant efficiency of 90%, after first computing the fuel efficiency of generation.

*Technical potential of installed capacity*: Maximum installed capacity for onshore wind, offshore wind, and solar PV is taken from the estimate of technical-social potential by Tröndle et al. [15].

*Technical potential of generation*: Maximum annual generation for onshore wind, offshore wind, and solar PV is taken from the estimate of technical-social potential by Tröndle et al. [15]. Maximum annual generation for biomass, biogas, and waste incineration is primarily taken from the JRC-ENSPRESO database [18] unless indicated otherwise. Maximum annual generation for hydropower is estimated from Hoes et al. [16]. Development locations with a potential of more or equal to 10MW are assigned as dams, and locations of less than 10MW are assigned as run-of-river plants. The development locations are then aggregated within country borders to estimate national generation potential for each application. Hydropower pumped storage potential is directly taken from Stocks et al. [17].

*Maximum annual load factor*: For onshore wind, offshore wind, and solar PV generation, original hourly time series from Renewables.ninja [18,19] are used directly as input to the model and are not redistributed in this data package. The LF_max variable in these technology files represents a placeholder value. For run-of-river and dam hydropower, annual load factors are estimated from ENTSO-E daily series [20], averaging across all days of the year to obtain an annual average load factor. Missing countries are replaced with the EU27 average and values are bounded to minimum and maximum observed historical load factors. Maximum load factors for other generation technologies are estimated from typical modeling practice.

*Minimum annual load factor*: Modeling assumption.

*Ramp rate:* Modeling assumption.

*Contribution of installed capacity to the peak load equation*: Modeling assumption.

*Investment cost*: Investment costs for onshore wind, offshore wind, and solar PV are primarily taken from IRENA [22]; missing countries are replaced with the average investment cost across all countries. Investment costs for fossil and nuclear plants are estimated from OECD/Nuclear Energy Agency [8]; missing years and countries are replaced with the average investment cost across all countries. Investment costs for other technologies are taken from EC-ASSET [23]. All values represent overnight investment costs unless specified.

*Fixed operation and maintenance costs*: Fixed costs are set to zero for fossil fuel-based and nuclear technologies, which instead assume levelized operation and maintenance costs consistently with OECD/Nuclear Energy Agency [8]. Fixed costs for other technologies are taken from EC-ASSET [23].

*Variable operation and maintenance costs*: Levelized operation and maintenance costs for fossil fuel-based and nuclear generation are estimated from OECD/Nuclear Energy Agency [8]. Missing years and countries are replaced with the average costs across all countries. Other technologies use variable costs from EC-ASSET [23].

*Technology learning rate*: Based on Rubin et al. [24]

*Maximum annual addition of generation capacity*: Modeling assumption.

*Lifetime of generation capacity*: Modeling assumption.

### 2.3 Resource Files

*Price of generation fuel or resource (per unit of fuel or resource)*: For oil, gas, and coal, values are primarily taken from IEA [25] unless indicated otherwise in the CSV files; alternative open-access references are provided when available. Missing countries are estimated using data for fuels imported to Germany. Fuel prices for biomass, biogas, and waste incineration are primarily taken from the JRC-ENSPRESO database [18]. Annual average imported electricity prices are estimated by dividing value of imported electricity (Eurostat dataset DS-018995, SITC code 35) by its volume (Eurostat dataset nrg_bal_c, balance IMP). Missing years are estimated using the average industrial electricity price in neighboring countries from Eurostat datasets nrg_pc_205_h and nrg_pc_205 (before 2007: price for demand band Ie, first semester; 2007 and later: band Id, first semester).

*CO_2_ emissions of generation fuel or resource (per unit of fuel or resource)*: CO_2_ emissions factors for fossil fuels are taken from IPCC guidelines [26]. National CO_2_ emissions factors for imported electricity are estimated as a weighted average of electricity imports from neighboring countries using Eurostat dataset nrg_ti_eh and European Environmental Energy grid emissions factors [27].

### 2.4 Load Profile Files

*Hourly load*: For 2000–2005, all available profiles were digitized from UCTE annual reports [4], covering hourly load values for the third Wednesday of each month and for the second or third Saturday and Sunday of each month. For 2006–2019, hourly load values were directly taken from ENTSO-E [5] and Open Power System Data [6], discarding days missing more than six hourly values, and years missing more than 180 days.

## Ethics Statements

The authors declare that this research did not involve human or animal subjects or any collection of data from social media platforms.

## CRediT authorship contribution statement

**Marc Jaxa-Rozen:** Data curation, Software, Writing – original draft, Conceptualization, Writing – review & editing. **Xin Wen:** Software, Validation, Conceptualization, Writing – review & editing. **Evelina Trutnevyte:** Funding acquisition, Supervision, Conceptualization, Writing – review & editing.

## Declaration of Competing Interest

The authors declare that they have no known competing financial interests or personal relationships that could have appeared to influence the work reported in this paper.
